# Conducting a Cost-Benefit Analysis of Transitional Care Programmes: The Key Challenges and Recommendations

**DOI:** 10.5334/ijic.4703

**Published:** 2020-02-21

**Authors:** Ke Xin Eh, Ian Yi Han Ang, Milawaty Nurjono, Sue-Anne Ee Shiow Toh

**Affiliations:** 1 Singapore Population Health Improvement Centre (SPHERiC), National University Health System, SG; 2 Saw Swee Hock School of Public Health, National University of Singapore, National University Health System, SG; 3 Regional Health System Office, National University Health System, SG; 4 Centre for Health Services Research and Policy Research (CHSPR), Saw Swee Hock School of Public Health, National University of Singapore, National University Health System, SG; 5 Yong Loo Lin School of Medicine, National University of Singapore, National University Health System, SG

**Keywords:** transitional care, post-discharge care, integrated care, cost-benefit analysis, health expenditures, mortality

## Abstract

Transitional care encompasses a range of services designed to promote care integration as patients transfer between different locations or different levels of care. Transitional care programmes have been proven to produce positive outcomes in reducing hospital readmissions and improving patients’ health outcomes. However, little is known about the benefits of the programmes on healthcare cost and the published results have been inconsistent. With increasing healthcare expenditures and limited public healthcare resources, cost-benefit analyses become paramount in informing healthcare resource allocation decisions. This perspective paper describes the approaches used in estimating the total costs of a bundle of transitional care services from an academic medical centre, identifies the key methodological challenges encountered in the process of cost-benefit analysis, and recommends potential solutions to tackle these challenges. By providing a comprehensive perspective on the methodological challenges, this paper encourages program evaluators to take these possible challenges into consideration for future cost-benefit analyses.

## Background

Transitional care encompasses a range of services designed to promote care integration as patients transfer between different locations or different levels of care [1]. With the aim of facilitating seamless transitions for older patients with complex illnesses from hospital to home setting, the National University Health System, Singapore, implemented transitional care programmes. These programmes comprised a bundle of transitional care services including education on self-management, reconciliation of medications, home rehabilitation sessions, and psycho-social support provided by a multidisciplinary healthcare team based in an academic medical centre and its community partners.

Literature has provided substantial evidence that transitional care can improve patients’ health outcomes and reduce hospital readmission rates [2,3,4]. However, the cost incurred in providing transitional care is not well understood, providing limited insights to inform decision-makers about the feasibility, sustainability, and scalability of implementing transitional care [5].

In our evaluation study [6], the number of lives saved was found to have improved [7]. Cost-utility analysis measures more aspects of health and well-being but was not appropriate without findings demonstrating improvements in health-related quality of life. Hence, cost-benefit analysis was a more appropriate analysis in the context of our study, which was investigating whether the benefits (of lives saved) of the intervention exceeded its costs. The number of lives saved was converted to monetary terms by multiplying with the Value of Statistical Life to understand the mortality benefits of the transitional care programmes relative to the costs incurred. The overall cost incurred by patients in the programmes was comprehensively estimated from both the health system perspective (i.e. healthcare utilisation and programme implementation costs) and societal perspective (i.e. productivity losses, transportation, and caregiving costs).

Using our evaluation as a case study, we outlined the approaches used in estimating costs in various categories, highlighted the challenges encountered in conducting cost-benefit analysis, and provide recommendations to tackle these challenges.

### Healthcare Utilisation Cost

To estimate the healthcare utilisation cost, the inpatient admission charges, emergency department attendance charges, and specialist outpatient clinics attendance charges for patients enrolled in the programmes and their matched controls were extracted from the healthcare administrative database. The control patients were matched 1:1 to enrolled patients based on the baseline demographics, number of comorbidities, and healthcare utilisation charges using propensity score matching. The healthcare utilisation mean charges were then compared between intervention and control groups using linear regression.

### Programme Implementation Cost

To estimate programme implementation cost, manpower and transportation costs incurred in providing transitional care services for the enrolment period were included. The frequency and total duration spent on providing transitional care services per patient were extracted from the time log recorded by the healthcare team. Manpower costs were calculated by multiplying the mean total time spent on each patient with the hourly norm cost salary of the healthcare team. Transportation costs incurred were estimated by calculating the costs of the average round-trip distance from hospital to patient’s home.

### Societal Cost

A cost questionnaire incorporating the various aspects of societal costs such as outpatient non-physician services (i.e. physiotherapy, and rehabilitation), non-conventional medicine, medical equipment, productivity cost, formal and informal caregivers, and community services was used in this case study. Patients were requested to recall their healthcare expenditures over the past three months. The outpatient non-physician services and community services costs were based on patients’ self-reported charges. The costs of non-conventional medicine and medical equipment were valued by applying the norm cost of each item. For productivity losses, informal and formal caregivers’ costs, the average occupational wages based on industry were extracted from the Ministry of Manpower, Singapore [8]. The cost questionnaire was part of a questionnaire packet that also measured other patient-reported outcomes using 5-Level EuroQol-5 Dimension (EQ-5D-5L), Patient Assessment of Care for Chronic Conditions (PACIC), and Patient Activation Measure (PAM-13) questionnaires.

## Key challenges

A key challenge in this study was the difficulty in obtaining comprehensive cost data for cost-benefit analyses. The healthcare system in Singapore consists of both public and private sectors and they often have discrete sets of electronic medical record databases for their own administrative purposes [9]. This has created fragmentation of electronic medical records that hinder the exchange of information and potentially impede the continuity of patient care. Hence, it was challenging to capture the complete healthcare utilisation charges without the integration and sharing of data across the public and private sectors especially when there could be significant cross-utilisation of services between different healthcare sectors in Singapore [10]. The lack of information on subsidies was also an obstacle in estimating the complete healthcare utilisation costs as patients treated in the public sectors received different types of healthcare financing schemes and subsidies [11]. Additionally, the total costs might not be fully attributed to the programmes as patients might receive other similar interventions concurrently.

The reliability of the data and outcomes of any study is dependent on the rigour of the data collection methods [12]. In this study, incomplete logging of data was found as a barrier for us to estimate the total programme implementation cost. The healthcare team who delivered the transitional care services did not document the frequency and duration of services provided at the level of specificity required for a cost-benefit analysis. This was not surprising as there was no established workflow or guideline in documentation built into the operations protocol ahead of time to facilitate the consolidation of cost data. Even if there was, the tough balance would have to be struck between being detailed, accurate, and in line with the frontline job priorities that the healthcare team is serving [13].

Self-reported questionnaires can be useful in collecting societal cost [14]. However, recruitment of patients not enrolled in the intervention programme as control group participants can be challenging. Self-reported societal costs are also subjected to self-reporting biases and recall inaccuracies that under or overestimate service utilisation [14,15]. Given the growing healthcare interventions targeted at the elderly populations, the inclusion of productivity losses of unpaid labours become paramount in cost-benefit analyses as these patients may still perform household work, volunteer work, and provide informal care in the society [16,17]. However, these were often omitted in cost-benefit analyses due to the lack of consensus on the measurements that allow the translation of such productivity losses into monetary values [16].

The Value of a Statistical Life is an estimate measurement on how much people are willing to pay to reduce the risk of death [18]. This is the only feasible method to estimate the mortality benefits in this study due to the constraints from the lack of available data. The cost-benefit analysis commenced after the programmes have started implementation, and thus it was not possible to go back in time to collect the required data. As there was no standard Value of a Statistical Life in Singapore, a proxy was generated using the Value of a Statistical Life from UK and adjusted to Singapore value with a ratio of Gross Domestic Product [19].

## Recommendations

One way to reduce the fragmentation of healthcare costs data, caused by silos of data collection, is to integrate the electronic medical record databases across both the public and private sectors. Synchronising the electronic medical record databases from different sectors can be challenging due to the existing Personal Data Protection Act that restricts cross-institutional data sharing without prior expressed consent obtained [20]. A centralised linked database would have to be established on a cross-institutional level to capture the complete healthcare utilisation charges incurred at all healthcare settings such as hospitals, general practitioner clinics, pharmacies, and traditional medicine clinics. For instance, in Estonia, a nationwide Electronic Health Record system that unified patients’ medical records across the country by patients’ identity was implemented to enable the exchange of information between all healthcare providers (i.e. pharmacies and laboratories) [21].

Incomplete data logging could threaten the reliability and validity of the results [22]. In this study, documentation of services provided during home visits was done on paper and transferred into the hospital database, which were likely susceptible to inaccuracies and incomplete data logging. To ensure accurate and complete data logging by the healthcare team, an automated data capture system that allows the duration and frequency of transitional care services provided to be captured automatically should be implemented. This could facilitate the data collection process, improve data quality, and allow data to be consolidated in a comprehensive and systematic fashion to be used for cost-benefit analysis [23].

To reduce self-reporting biases and recall inaccuracies, appropriate designing of data collection methods, recall time frame, and mode of data collection should be done at the developmental stage of the programme [24]. Alternatively, a cost diary that requires patient to make regular records of the services received is particularly useful as events would be recorded as they occur and thus less likely to be affected by recall inaccuracies [25]. The lack of consensus in measuring productivity costs have likely contributed to the disregard of productivity losses from a societal perspective [16,17]. Future research should focus on developing a guideline that allows the translation of unpaid labours productivity losses into monetary values that is tailored to the local context.

While most of the studies investigating the Value of a Statistical Life have been conducted in many developed countries, no such study has been conducted in Singapore [19]. Future research should also focus on developing a Value of a Statistical Life in Singapore. Future work could also expand to investigating other benefits that transition care programmes could impact, such as quality of life as measured by EQ-5D-5L. The EQ-5D-5L values could be used to calculate quality-adjusted life years (QALYs) saved in cost-utility analyses.

## Conclusion

Using a case study, we have highlighted some of the lessons learned in conducting a cost-benefit analysis of transitional care programmes in Singapore. To obtain informative cost data for analysis, all the inclusion and exclusion components required for a cost-benefit analysis should ideally be well defined at the start of the programme. A better understanding of possible challenges, the pros and cons of approaches to address them, and their implications, can provide guidance for future cost-benefit analyses.
